# Learned Cardiac Control with Heart Rate Biofeedback Transfers to Emotional Reactions

**DOI:** 10.1371/journal.pone.0070004

**Published:** 2013-07-23

**Authors:** Nathalie Peira, Gilles Pourtois, Mats Fredrikson

**Affiliations:** 1 Department of Psychology, Uppsala University, Uppsala, Sweden; 2 Department of Experimental Clinical and Health Psychology, Ghent University, Ghent, Belgium; The University of Queensland, Australia

## Abstract

Emotions involve subjective feelings, action tendencies and physiological reactions. Earlier findings suggest that biofeedback might provide a way to regulate the physiological components of emotions. The present study investigates if learned heart rate regulation with biofeedback transfers to emotional situations without biofeedback. First, participants learned to decrease heart rate using biofeedback. Then, inter-individual differences in the acquired skill predicted how well they could decrease heart rate reactivity when later exposed to negative arousing pictures without biofeedback. These findings suggest that (i) short lasting biofeedback training improves heart rate regulation and (ii) the learned ability transfers to emotion challenging situations without biofeedback. Thus, heart rate biofeedback training may enable regulation of bodily aspects of emotion also when feedback is not available.

## Introduction

Emotion reactions that are part of everyday life involve subjective feelings, action tendencies and physiological reactions. In some situations, emotions need to be regulated in order to foster goal-directed behavior, and different strategies can be used to achieve this goal. Emotion regulation strategies are differentially successful in regulating the emotional reaction in terms of subjective feelings and physiological reactions [1]. For example, it has been found that when suppression is used as a regulation strategy, the physiological component is not decreased, but may even be increased [2]. It has also been suggested that unregulated physiological reactions increase the long-term risk for cardiovascular disease [3]. Thus, it is important to develop regulation strategies that could be implemented especially to decrease the physiological reactions that form part of the emotional complex.

Biofeedback stands out as an interesting strategy given its propensity to act and alter ongoing physiological reactions [4]. Supporting its clinical relevance, it has been shown that patients with anxiety disorders may experience symptom reductions with biofeedback training [5], [6]. In this context, biofeedback training appears valuable because it could be used as an emotion regulation strategy selectively targeting the physiological reaction elicited by an emotional stimuli or situations.

In a previous study [7], we showed that heart rate biofeedback during exposure to negative pictures can be used to regulate the physiological reactions elicited by these stimuli. Although promising, these results raise the question whether the use of biofeedback is dependent on the immediate feedback availability or whether the participant develops a skill during feedback training that transfers to situations without feedback. If no such transfer effect could be shown, an obvious drawback would be that biofeedback would require the presence of a biofeedback device. Conversely, a clear advantage of biofeedback would be if individuals acquired a skill that could be applied to regulate physiological reactions in situations without feedback, as when confronted with negative emotion.

There is some evidence suggesting that training with biofeedback may transfer to other situations in which feedback is no longer given. For example, stress induced heart rate reactivity was reduced with biofeedback training and transfered from training to a stressful laboratory task (mental arithmetic task) in which no feedback was given [8]. However, participants were not only trained with biofeedback but also informed about visualization, breathing and relaxation techniques to control heart rate and explicitly invited to practice those at home when no feedback was available. Thus, because participants practiced to control heart rate with several techniques without biofeedback, it is not possible to dismantle the effect specific for biofeedback.

If heart rate control to stress can transfer from training with biofeedback to tasks without biofeedback, then heart rate control might show the same transfer effect to exposure to other emotionally challenging situations like negative arousing pictures. However, there is a difference in how heart rate responds to negative arousing pictures as compared to stress. Responses to negative arousing pictures are multiphasic. After an initial deceleration (i.e. orienting response), heart rate accelerates and later decelerates again [9], [10]. Although arousing pictures elicits both heart rate accelerations and decelerations, participants in the present study were instructed to decrease heart rate, for two reasons. First, because a previous proof of concept study demonstrated that participants were able to systematically decrease their heart rate during biofeedback when asked to do so when exposed to negative arousing pictures [7]. Second, because heart rate accelerations are prominent in specific phobia, social anxiety, post-traumatic stress disorder and panic disorder [11], and as symptomatic treatment reduce heart rate, we hypothesized that training to decrease heart rate would result in a reduction in the experienced negative affect.

The aim of the present study was to investigate if the skill acquired as a function of biofeedback training will transfer to heart rate control without feedback during a negative affect challenge.

## Methods

### Ethics Statement

The study was approved by the local ethics committee (Faculty of Psychology – Ghent University) and conducted in accordance with the declaration of Helsinki. Participants were informed about the voluntary nature of participation, signed an informed consent form prior to the experiment, and were fully debriefed about the purpose of the study at the end of the experiment. No participants were under the age of 18.

### Participants

Twenty undergraduate students from Ghent University were recruited through an internet based recruitment portal (age: M = 22.40, SD = 4.84). There was no age difference between men (N = 6, age: M = 24.50, SD = 7.18) and women (N = 14, age: M = 21.50, SD = 3.39) (t >1, p>0.05). Participants were given 8 euros for participating in the experiment that lasted about 1 hour.

### Apparatus and Materials

#### Set up

The experiment was conducted in a sound-attenuated room. Pictures were presented at a distance of 0.6 m on a cathode ray-tube (CRT) monitor (21 inches, 1024×768 pixels resolution) with software written in Presentation 10.3 (Neurobehavioral Systems, www.neurobs.com). Electrocardiography (ECG) was recorded with a Biopac MP150 system with a sampling rate of 200 Hz in standard lead II configuration: The right arm electrode was placed near the right collarbone, and the left and right leg electrodes on the right and left side of participants’ ribcage. Heart rate was calculated online with Acqknowledge software. For triggers and heart rate feedback, the experiment computer and the computer with Acqknowledge software were connected with a parallel port. Also skin conductance was measured but due to equipment failure in most participants data was not possible to evaluate.

#### Picture material

Twenty negative pictures were selected from the international Affective System (IAPS) [12] based on the normative ratings provided with this picture set. Negative pictures (arousal between 6.3 and 10, valence between 3.8 and 1.7) were pre-selected in such a way to include as many fear related pictures as possible and avoid mutilations because these are related to disgust responses and as such general deceleration in heart rate [13]. Pictures were 1024×768 pixels and scaled to 0.7 times the size in Presentation software. The pictures were, for each participant, randomly assigned to either pre- or post-test. Thus, the pictures were never the same for the pre- and the post-test for any participant. Also, because picture assignment was done separately for each participant, as a result, the pictures shown during the pre- and post-test were fully randomized.

#### Biofeedback

The feedback reflected participant’s actual heart rate changes and was presented in the form of background color changes on the screen. In the Acqknowledge software of the Biopac module, heart rate was computed online and was monitored by a calculation channel. When heart rate changed more than 0.1 bpm, the calculation channel sent a signal through the parallel port to the Presentation computer. The presentation software monitored the parallel port and updated the color of the screen accordingly every 500 ms. If heart rate had accelerated the color was changed towards red while if it had decelerated it was changed towards green. The color change was made by adjusting the red and green values of the RGB of the screen by 40 steps (the values of the RGB each ranged from 0 to 255). Each trial started at yellow (R = 255; G = 255; B = 0). To turn the screen more red, the G value was decreased. To turn the screen more green, the R value was decreased.

#### Questionnaires

Participants’ general anxiety was assessed with the trait version of the State-Trait Anxiety Inventory (STAI) [14] based on 20 statements on a 4-step scale. Participants’ emotion awareness was assessed with the Toronto Alexithymia Scale (TAS) [15] based on 20 items on a 5-point scale, and the Emotion Awareness Questionnaire (EAQ) [16] based on 30 items on a 3-point scale. Participants’ emotion regulation strategies was assessed with the Emotion Control Questionnaire (ECQ) [17] based on 56 true/false items with four subscales (rumination, inhibition, aggression control, beneficial control), and the Emotion Regulation Questionnaire (ERQ) [18] based on 10 items on a 7-point scale with two subscales (suppression and reappraisal). These questionnaires were all administered in Dutch, except the ECQ (English).

### Procedure

#### General

**Figure 1:**
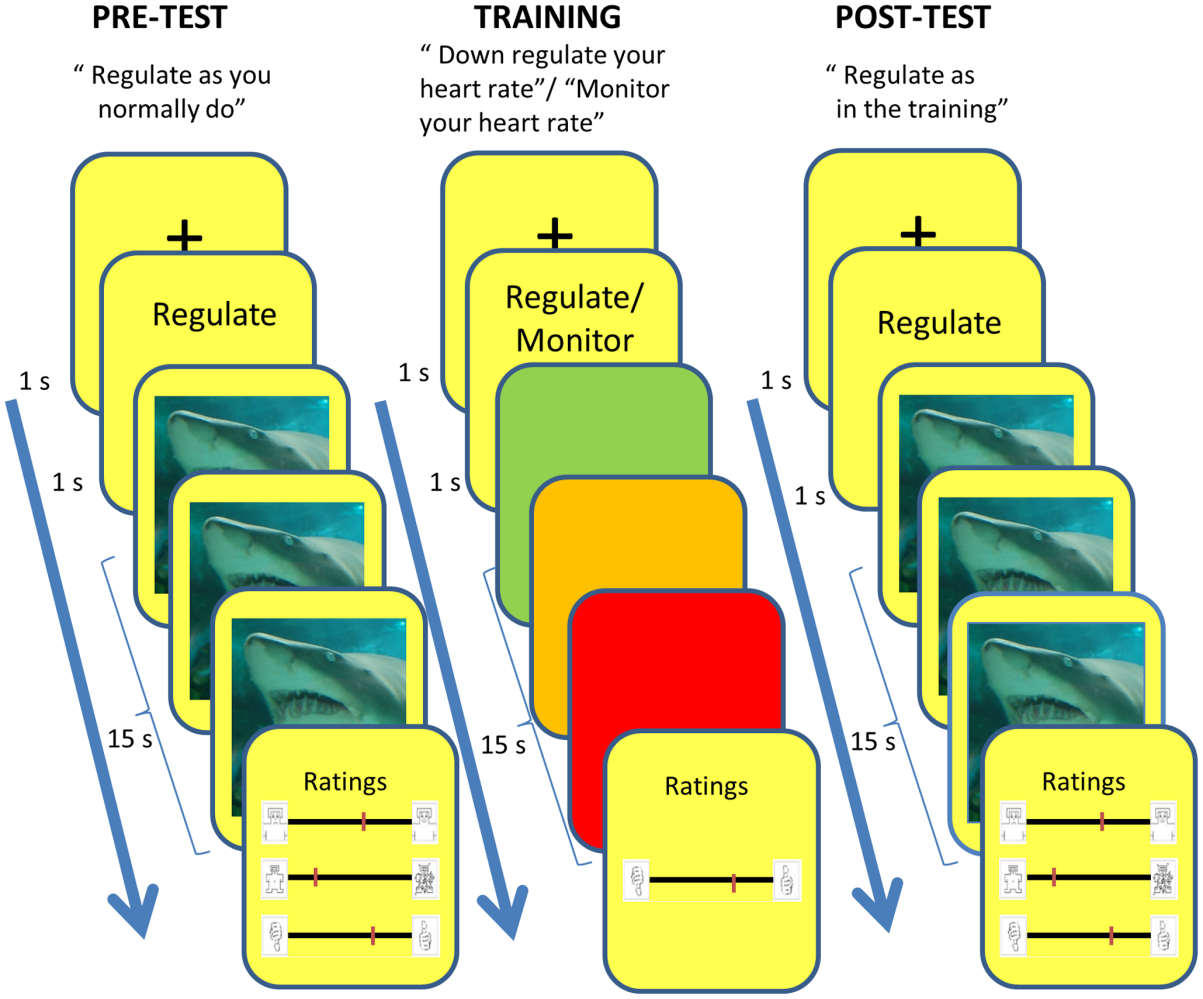
Example of training, pre- and post-test trials.

The experiment was designed as a pre-post training experiment. During the training participants only received color heart rate feedback on the screen and no pictures were displayed. During the pre- and post- tests participants were exposed to negative pictures and never received any heart rate feedback (see figure 1). After the task, participants completed computerized versions of the questionnaires.

#### Pre-test

In the pre-test, participants were exposed to ten trials of negative pictures and never received heart rate feedback. Participants were instructed to regulate their emotions as they normally do, without any further instructions. Each trial consisted of a fixation cross (1 s) and an instruction to regulate (1 s) followed by a negative picture for 15 s, and ended with ratings. This stimulus presentation duration was chosen as to enable room for changes in the heart rate after the initial phasic (orienting) response to the picture onset. Participants rated how they felt during the picture viewing (valence and arousal) and how successful they were in performing the task. The ratings were performed on a continuous scale by moving the mouse from one end of the screen to the other with the most extreme figures from the Self-Assessment Manikin (SAM) [19] shown at the two opposite anchors of the scale. For the success ratings, the start and end figures were schematic thumbs-up and thumbs-down. To keep participants motivated to look at the picture, a question on the picture content (i.e. if the scene was outdoor or indoor) was presented on 1/10 of the trials (i.e. catch trials).

#### Training

During the training, participants received feedback about their heart through color changes on the screen. Each trial consisted of a fixation cross (1 s) and a short task instruction (1 s) followed by heart rate feedback for 15 s, and at the end subjects completed a task success rating. Participants had two tasks. Half of the trials were active training trials with the instruction to down regulate heart rate, and the other trials were control trials with the instruction to monitor heart rate. The two tasks were presented randomly within blocks of four trials (i.e. 2 regulate and 2 monitor heart rate). These four-trial blocks were repeated 15 times (i.e. in total 60 trials). During the regulate trials participants were instructed to try different strategies to lower their heart rate, discard what did not work and keep on doing whatever worked. It was emphasized that it was strictly forbidden to affect heart rate during the monitor trials and to hold the breath during any trials (controlled breathing was allowed).

#### Post-test

In the post-test, the pre-test procedure was repeated but with the instruction to regulate the reactions to the pictures with the strategies that were effective during the training. At the end of the experiment, participants filled in computerized versions of the questionnaires.

### Data Processing

#### Data screening

Heart rate measures were scanned for artifacts using 3 criteria. First, heart rates above 150 or below 40 bpm were discarded. Second, heart rates with a difference bigger than 35 bpm within a time window of 1000 ms were discarded. Third, each sample point was compared to a sample point 100 ms before. The sample point was discarded if the difference was bigger than 35 bpm.

#### Baseline correction

Heart rate was computed as the heart rate median in 1 second intervals resulting in 15 bins for each trial. Heart rate measures were baseline corrected to the four seconds immediately before picture onset.

#### Quantification of the training effect

To rule out that time or habituation effects were mislabeled as training effects, training was quantified as a slope fitted to the successive difference between active training (regulate HR) and control (monitor HR) trials. These active training and control trials were equally distributed over the training session (with repetition of blocks of 2 regulate and 2 monitor HR trials). For each block, a difference between the active training and control trials was calculated (regulate HR – monitor HR). A line (linear function y = ax+b) was fitted to the successive block differences. Because habituation, if present, likely occurs at an equal rate during both trial types, the slope of the training was based on the blockwise difference between the two trial types. Thus, habituation effects was experimentally controlled within each individual. With this method, a successful training is reflected in a larger heart rate decrease in regulate as compared to monitor trials over the training, yielding a negative slope. The slope value (a) was inverted to reflect performance gains with bigger training effects resulting in more positive values. Thus, the training effect can be described as the increase in the difference between regulate and monitor trials from start to end of training.

## Results

### Training

Participants successfully learned to control heart rate during the training, as reflected by a positive slope significantly different from zero (m = 0.18, sd = 0.33; t(19) = 2.472, p = 0.023). Thus, as a function of training participants were able to decrease heart rate increasingly more in the regulate compared to the monitor trials. To investigate if the training effect on heart rate was paralleled by the success ratings, the difference in success ratings between regulate and monitor trials over time was computed in the same way as the training effect (i.e. the slope (a) of the individually fitted linear function y = ax+b). The slope of the success ratings correlated with the training effect (r = −0.516, p = 0.020, r^2^ = 0.26). This suggests that the larger the training effect, the more the difference in success ratings between regulate and monitor trials decreased as a function of training. Thus, participants with a larger training effect tended to rate the regulation success as increasingly more similar to the success of the easier task consisting of just monitoring their heart rate.

### Pre-post Training Differences

**Figure 2:**
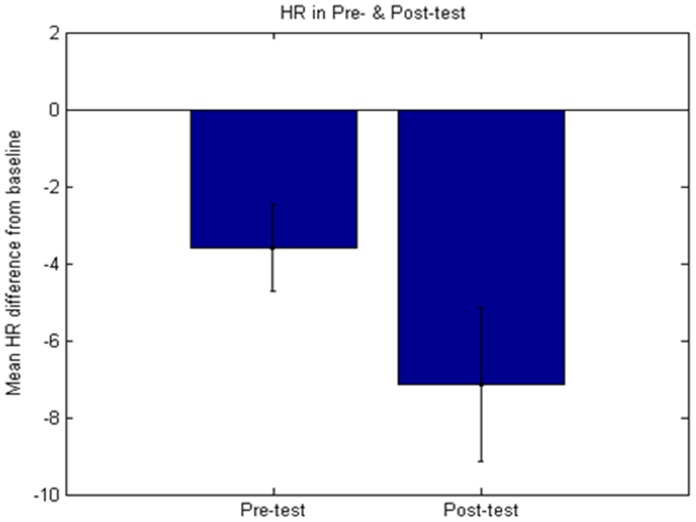
Heart rate differences in bpm from baseline in the pre- and post-test. Error bars represent 95% confidence intervals (note that the error bars reflect between and not within subject variance and as such are non-informative for the within subject statistical tests used).

Participants displayed lower heart rate in the post-test (m = −7.1) compared to the pre-test (m = −3.6; F(1,19) = 13.12; p = 0.002, η_P_^2^ = 0.41) demonstrating that they were better at regulating heart rate to negative pictures after training (see figure 2). Ratings of task success (p = 0.628), valence (p = 0.111) and arousal (p = 0.826) did not show significant pre-post differences. Pre-post differences in rated task success correlated with pre-post differences in rated valence (r = 0.664, p = 0.001) and arousal (r = −0.435, p = 0.055; trend) but did not correlate with the pre-post difference in heart rate (all p>0.332).

### Transfer of Training

**Figure 3:**
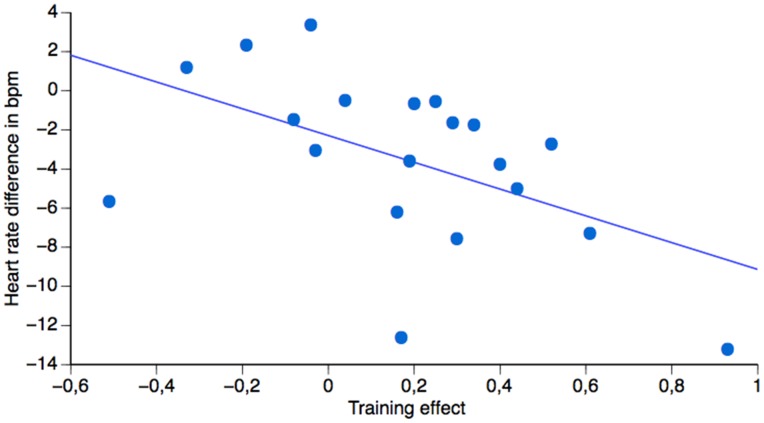
The biofeedback induced skill transfers to emotional challenging conditions with no feedback. Correlation between the training effect (X-axis) and the pre-post training difference in heart rate (HR) (Y-axis). For the training effect, a more positive value indicates a larger decrease in heart rate when regulating compared to monitor as a function of training. For the pre-post training difference (Y-axis), a more negative value corresponds to a larger decrease in heart rate post- as compared to heart rate pre-training.

To specifically evaluate the prediction that biofeedback training transfers to conditions when feedback no longer is available and when emotion challenging pictures are presented, we analyzed if the individual differences in training performance correlated with pre-post differences in heart rate. Results showed a relatively high correlation between the training effect and pre-post heart rate differences (r = 0.518, p = 0.019, r^2^ = 0.27). Thus, the better the training effect, the larger was the decrease in heart rate from pre- to post-training (see figure 3), confirming that performance differences during training accounted for the pre-post difference in heart rate regulation. The training effect did not correlate with pre-post differences in task success (p = 0.740), valence (p = 0.445) and arousal (p = 0.867), suggesting a specific physiological learning effect without a corresponding change in experience.

### Questionnaires

Participants’ general anxiety (STAI), emotion awareness (TAS & EAQ), and emotion regulation strategies (ECQ & ERQ) were measured. We assessed whether these scores correlated with the training effect, and the pre-post difference in heart rate. These analyses revealed that only two of the emotion regulation strategies (from the ECQ) correlated significantly with both the training effect and the pre-post difference in heart rate (all other ps >0.05). Specifically, aggression control correlated positively with the training effect (r = 0.799, p<0.001) and the pre-post difference in heart rate (r = −0.497, p = 0.042). Moreover, rumination correlated negatively with the training effect (r = −0.527, p = 0.030) and the pre-post difference in heart rate (r = 0.577, p = 0.015).

## Discussion

The results of the present study showed that (i) short lasting biofeedback training improves heart rate regulation and (ii) the learned ability transfers to emotion challenging situations without biofeedback. Thus, the better participants learned to regulate heart rate with biofeedback, the better they were at applying that skill when later presented with negative arousing pictures, even though feedback was no longer available.

Heart rate regulation improved during training reflected in an increased within-subject difference between active training trials (with the instruction to regulate HR) and control trials (with no instruction to regulate but only to monitor HR) over the training session. The design controls for effects of habituation and time for each participant as active training and control trials were equally distributed over the training session (with repetition of blocks of 2 training and 2 control trials) and the performance estimate (the slope) was based on the difference between the trial blocks. This within-subject design is sensitive to reveal learning effects given that large between-subject variability in the physiological response might mask small learning effects.

The learned skill to decrease heart rate transferred to the emotion challenge after training, even though feedback was not presented. This was reflected in a relatively high correlation between individual differences in training performance and pre-post differences in heart rate. This shows that the improvement in the ability to decrease heart rate acquired during training influenced heart rate regulation performance after training. However, some limitations on the generalizability of the results should be noted. Participants only viewed negative pictures in the pre- and post-tests. As such, we cannot conclude if the improved heart rate regulation is specific to negative emotions or if it would transfer also to positive emotions.

Results from the questionnaires suggest that habitual use of rumination may interfere with the deployment of an efficient emotion regulation strategy during biofeedback [20]. However, emotion awareness and general anxiety levels did not show any effects on how well an individual can learn to use biofeedback. The latter finding may indicate that individuals with high levels of trait anxiety and/or emotion awareness deficits also could presumably benefit from heart rate biofeedback training. However, a word of caution is needed regarding the interpretation of all null findings, given our modest sample size. Interestingly, the fact that rumination did correlate with the training effect suggests that we actually had sufficient power to detect significant correlations between changes in psychophysiology and questionnaire data.

During training, participants’ ratings of task success reflected task performance. But when applying the learned ability, participants’ ratings of task success did not correlate with task performance. During training, participants could use the feedback to receive correct information on how they succeeded in decreasing heart rate. Thus, it is not surprising that participants’ ratings of task success correlated with actual success when learning to decrease heart rate during the training. However, during the pre-posttests feedback was no longer available and there was no correlation between rated and actual task success. Also, pre-post differences in ratings of valence and arousal did not show any correlation neither with the pre-post differences in heart rate, nor with the heart rate training effect. Thus, participants learned to decrease heart rate during training, the learned skill transferred to exposure to negative pictures even though feedback was no longer available, but this did not affect participants’ experience of the pictures or of task success. Our results show that heart rate, but not participants’ subjective experience, was better regulated after the short exposure to a biofeedback session (post-test), compared to what they did at baseline (pre-test) using a spontaneous or habitual regulation strategy.

The decoupling of heart rate decreases from the experience of success, valence and arousal suggest a dissociation between body and experience. Note that this dissociation cannot reflect a failure in the assessment of the ratings because rated task success during training correlated with the heart rate training effect. Instead, the discrepancy between physiology and experience might be explained by a lack of awareness of the change in heart rate. That is, participants did not feel or sense consciously that they actually decreased their heart rate. If the difference in heart rate is not consciously perceived, it might not be appraised and taken into account in the evaluation of the emotional experience, which typically requires conscious access. In agreement with this conjecture, earlier results have shown that awareness of bodily states can influence the intensity of the perception of emotions [21]. We believe that awareness of the heart rate changes might be a crucial component. Participants’ awareness of an attenuated heart rate response was probably absent, preventing participants to relate heart rate to their subjective experience or evaluation of the pictures. Hence, future studies could try to increase subject’s awareness of the heart rate changes in order to assess whether this might lead to an altered subjective evaluation of the picture content after learning.

In summary, our results show that participants can learn to regulate heart rate in one context during a short biofeedback training and apply the learned skill in a different emotionally challenging context also in the absence of feedback. This implies that participants can learn to cope with emotionally induced physiological reactions and to attenuate their deleterious impact on the homeostasis. The long term effects may include cardiac protection.
